# *Oenanthe incrassans*: An enigmatic species from Turkey and its comparison with *Oenanthe pimpinelloides* (Apiaceae)

**DOI:** 10.3897/phytokeys.62.8106

**Published:** 2016-04-15

**Authors:** Ebru Doğan Güner

**Affiliations:** 1 Health Services Vocational School, Gazi University, 06830, Ankara–Turkey

**Keywords:** Anatomy, micromorphology, pollen, Oenanthe, taxonomy, Turkey

## Abstract

*Oenanthe incrassans* (Apiaceae) was discovered in Istanbul, Turkey. It is related to *Oenanthe pimpinelloides*, but it clearly differs in terms of leaves, inflorescence (ray, bracts, and bracteoles) and fruit features. A taxonomic description, some photographs of the species, geographical distribution and habitat features are given. Additionally, fruit micromorphology, stem, ray and fruit anatomy, and pollen features are studied for the first time and compared to *Oenanthe pimpinelloides*.

## Introduction

*Oenanthe incrassans* Bory & Chaubert (Bory 1832) is one of the synonyms of the *Oenanthe pimpinelloides* L. (Linneaus 1753) in Flora of Turkey (Hedge and Lammond 1972). Foley and Southam published a study on *Oenanthe incrassans* and they recognized it as a distinctive plant of the Aegean region (Foley 2007). The species is an element of the East Mediterrenean area because of its distribution in Aegean region, but it hasn’t been collected from Turkey until recently.

Foley and Southam also discussed *Oenanthe thracica* Griseb. which is the other synonym of *Oenanthe pimpinelloides* (Hedge & Lammond, 1972). They said that *Oenanthe thracica* is conspecific with *Oenanthe pimpinelloides* and its taxonomy is in need of further study. Their result was based on examination of a specimen of *Oenanthe thracica* recorded as “Turkey (European)–A1(E) Edirne: Kesan, 6 July 1982, Nydegger 17003”. In 2013, Özhatay et al. erroneously reported *Oenanthe incrassans* as a new record for Flora of Turkey based on this record (Özhatay 2013), and then it was added in the list of Flora of Turkey as a “doubtful species"(Menemen 2012). I have also examined specimen of Nydegger (deposite in E) which Foley and Southam determined as *Oenanthe thracica*, and in my opinion it is definetely *Oenanthe pimpinelloides*. This is consistent with an earlier determination of the sheet by Huber-Morath. Menemen (2012) thus recognized nine species (including doubtful species) in Turkey.

Within the scope of revisionary studies on the *Oenanthe* species in Turkey, numerous field trips were held between 2014–2015, on one of which *Oenanthe incrassans* was discovered in Istanbul. Additionally, W and WU herbarium in Vienna, Austria were visited to investigate specimens of *Oenanthe*. During the investigation, undetermined specimens which were collected by Ernst Vitek from Istanbul were identified as *Oenanthe incrassans*.

This study aims to present a full description of the species and resolve the delimitation between *Oenanthe incrassans* and *Oenanthe pimpinelloides* by comparing morphological, anatomical, palynogical and micromorphological analyzes and their ecological features.

## Methods

The specimens of *Oenanthe incrassans* and *Oenanthe pimpinelloides* were collected in different regions of Turkey between 2014–2015 and checked with relevant literature (Hedge and Lammond 1972, Cook 1981, Duman 2000). Herbarium specimens were deposited at GAZI. The specimens were compared with the types and other representative collections present at E, W, WU, GAZI (abbreviations following Thiers 2016). For the anatomic analysis, stem, ray, and fruit parts of the collected specimens were kept in 70% alcohol. Hand-made cross sections were firstly stained in sartur reagent (Çelebioğlu 1949). Detailed anatomic structures of the cross sections were photographed with a stereo microscope attached with a camera (Olympus E330). Relevant resources were made use of during the anatomic evaluation (Mauseth 1988, Dickison 2000).

Pollen acquired from anthers of the herbarium specimens were prepared based on Wodehouse method, stained with basic fuchsin, and analyzed under light microscope (Wodehouse 1935). The pollen samples were placed on aluminium tape, coated with gold by using Polaron SC 502 Sputter Coater device, and microphotographed by Jeol JSM 6490LV model scanning electron microscope (SEM). SEM analysis of mericarp micromorphology was conducted with the same method. The terminology of the pollen and mericarp is based on Moore et al. 1991, Punt et al. (2007), and Doğan Güner et al. (2011).

## Results

### Oenanthe incrassans

Taxon classificationPlantaeApialesApiaceae

Bory & Chaubert, Exp. Sci. Moreé, Bot.:87. 1832.

Figs 1 , 2 , 3 , 4 , 5 , 6 , 7 , 8 , Table 1 , 2

≡Oenanthe incrassata Bory & Chaub. in Chaub. & Bory, Nouv. Fl. Pelop.: 19. 1838

#### Lectotypus.

Bory & Chaub., Exp. Sci. Moreé, Bot.: tab. 8. 1835. (designated by Foley 2007!)

#### Specimens examined.

**GREECE. Crete**: Listr. Malevyzi, in paludosis fluviorum Gazanos et Almyros prope Gazi, 25 June 1942, KH. Rechinger fil. 14050 (W!); Sphakia: Sumpfiger Badem bei Frankokasteli, 13 April 1904, I.Dörfler, (WU!); **Corfu**: Ipsos to Ag. Markos, 16 July 1972, sides of moist fields, Davis 54531 (E); in einem Sumphe unterhalb des königlichen Schlosse Monrepos, 9 May–4 June 1996, Baenitz s.n. (E!); Ex regione collina Insula Corcyra, June 1877, Ball s.n. (E!); **Ep. Milopotamas**: b. Murdzana am N–Fuss der Kulukunas–Berge, 18 April 1962, W. Greuter 4170 (W!); **Cephalonia (Argostolion)**: Chelmata–Kompothekrata region, 15 April 1967, E. Stamatiadou 207 (W!); **Kissamos**: lieux humides, 2 May–2 July 1884, Reverchon 247 (as Oenanthe callosa) (E!); **Thasos**: Limenas, 19 May 1891, Sintenis & Bornmüeller 451 (W!);

**TURKEY. Istanbul**: c. 35 km NW von Istanbul, bei Durusu, am Ufer des Durusu–Sees, 20 m s.m., 41°17'43"E/ 28°35'40"N, 16 May 2000, E. Vitek 2000–28 (W!); Terkos to Karaburun, 20–50 m, marshy lakeside, 30 May 2014, ED. Güner 2009 (GAZI); ibid. 15 June 2015, ED. Güner 2098 (GAZI).

Perennial, 50–70 cm tall, herb, glabrous, with ovoid or oblong tubers far from stem base. Stem erect, sparsely branched above, hollow, deeply striate (furrowed). Basal and lower stem leaves 2–pinnate, ovate to lanceolate in outline, up to 15 cm with petiole; ultimate segments with pinnatifid lobes, ovate, 9–15 × 8–14 mm; petiole shorter than leaf lamina, broader at leaf base. Upper stem leaves 2–pinnate, ovate-triangular in outline; ultimate segments 2–2.5 cm long and 2–5 mm broad, elliptic. Umbels with 7–12 rays of subequal length (1.5–2 cm), rays becoming hardly thickened and elongating in fruit; involucral bracts 0–1, linear, up to 6×1 mm. Umbellets almost flat, with unequal, thickened pedicels in fruit, many flowered, about 1.5 cm diam., pedicel of surrounding flowers longer than inner ones. Bracteoles 10–12, linear, ca. 3 × 1 mm. Petals radiating, creamy white, the outer flowers are female, petals cordate, deeply emerginate in tip, inner petal surface papillate. Styles shorter than fruit, fruit oblong, 3.5–4 × 2–2.5 mm.

#### Distribution, habitat and ecology.

*Oenanthe incrassans* is distributed in Greece and Turkey (Figs 2–3). The species is distributed around Istanbul (Arnavutköy, Durusu–Terkos region), Turkey. The flowering time is April, fruiting time is June. It grows on lake sides at 20–50 m altitude.

**Figure 1.**
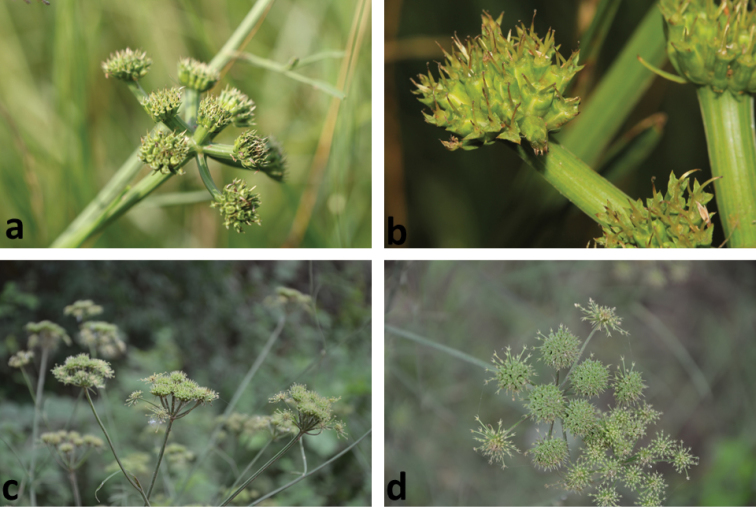
General view of inflorescens of **a–b** *Oenanthe incrassans* **c–d** *Oenanthe pimpinelloides*.

**Figure 2.**
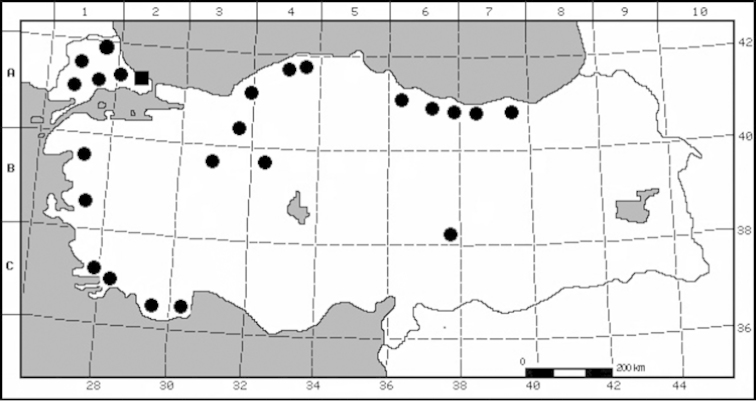
Distribution map of the *Oenanthe incrassans* (■) and *Oenanthe pimpinelloides* (●).

**Figure 3.**
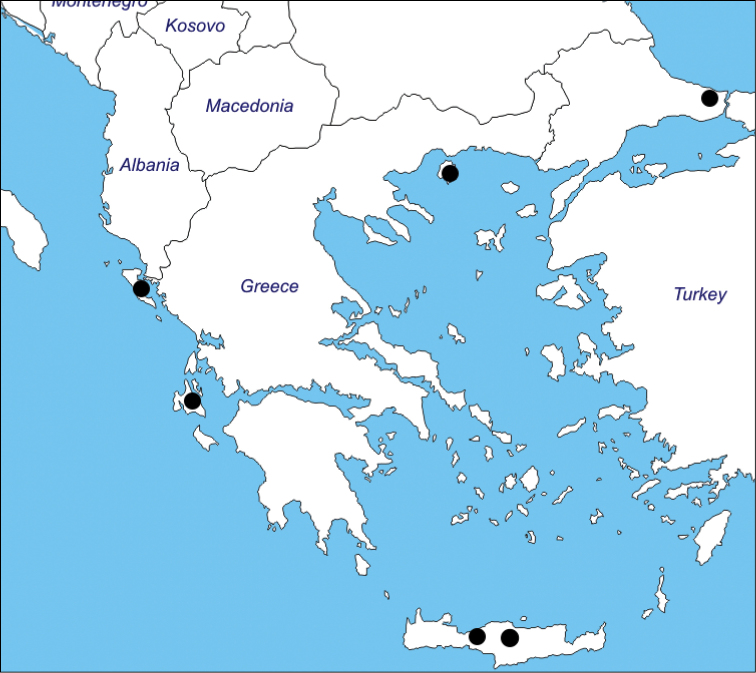
Distribution of the *Oenanthe incrassans* (●) in Greece and Turkey.

#### Morphology.

*Oenanthe incrassans* is close to *Oenanthe pimpinelloides*, but it clearly differs in leaves, inflorescens and features of fruit. Their differences are given in Table 1.

**Table 1.** Comparison of the morphological characters of *Oenanthe incrassans* and *Oenanthe pimpinelloides*.

	*Oenanthe incrassans*	*Oenanthe pimpinelloides*
Ultimate segments of basal and lower stem leaves	Pinnatifid, ovate, 9–15 × 8–14 mm	Pinnatilobate or pinnatifid, ovate–triangular, 8–10 × 5–8 mm
Ultimate segments of upper stem leaves	Elliptic, 20–25 × 2–5 mm	Linear or narrowly elliptic, 30–35 × 0.4–1.5 mm
Rays and pedicels	Strongly thickened	Thickened
Bracts	0–1	0–3
Bracteoles	10–12, ca 3 × 1 mm	12–14, 1.5–2 × 0.5 mm
Sepals	0.4–0.9 mm in fruit	0.2–0.4 mm in fruit
Styles	Shorter than fruit	± Equal fruit body
Fruit	3.5–4 × 2–2.5 mm	2.5–3 × 1–1.5 mm

#### Anatomy.

**Figure 4.**
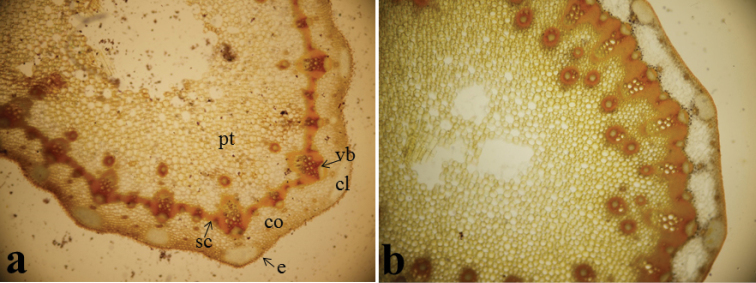
Cross sections of stem (10 × 5), **a** *Oenanthe incrassans* **b** *Oenanthe pimpinelloides*, (cl: collenchyma, co: cortex, e: epidermis, pt: pith, sc: sclerenchyma, vb: vascular bundle).

*Stem anatomy*: The shape of stem cross section is triangular or ovoid in outline in *Oenanthe incrassans*; whereas it is circular in *Oenanthe pimpinelloides*. Parenchymatic cells of cortex 4–5–seriate in *Oenanthe incrassans*; but it is 2–4–seriate in *Oenanthe pimpinelloides*. Sclerenchyma tissue cells are 4–5–seriate between two peripheral vascular bundles in *Oenanthe incrassans*; while they are 10–12–seriate in *Oenanthe pimpinelloides*. 1–2 small central bundles are placed below peripheral bundles in *Oenanthe incrassans*; but 1–3 central bundles are placed below peripheral bundles in *Oenanthe pimpinelloides* (Fig. 4a–b).

#### Ray anatomy.

**Figure 5.**
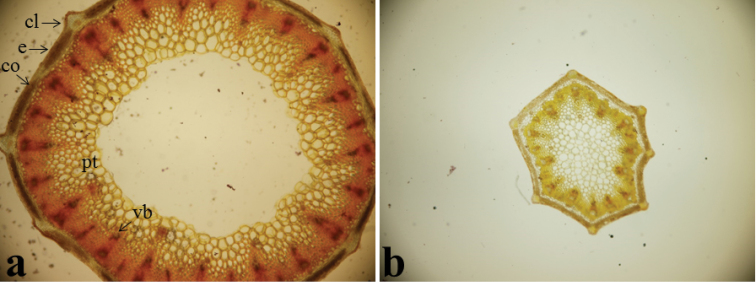
Cross sections of ray (10 × 5), **a** *Oenanthe incrassans* **b** *Oenanthe pimpinelloides*, (cl: collenchyma, co: cortex, e: epidermis, pt: pith, sc: sclerenchyma, vb: vascular bundle).

Rays are hardly thickened and the shape of cross section is 8–10–ridged and circular in outline in *Oenanthe incrassans*; but they are slightly thickened and 7–ridged ovoid or oblong in outline in *Oenanthe pimpinelloides*. There are 8–9–seriate collenchyma cells in *Oenanthe incrassans*; but 5–6 seriate in *Oenanthe pimpinelloides*. Pith cells are 3–4–seriate and disappear towards the center in *Oenanthe incrassans*; but they are present at the center in *Oenanthe pimpinelloides* (Fig. 5a–b).

#### Fruit anatomy.

**Figure 6.**
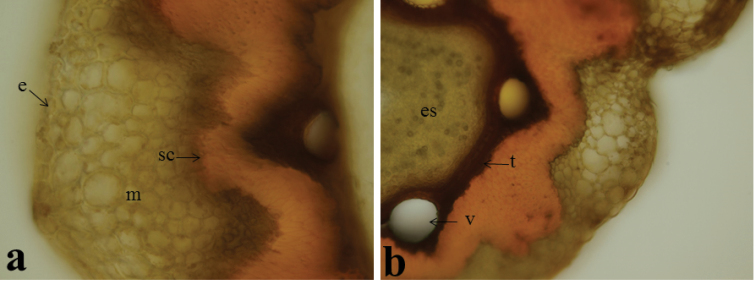
Cross sections of mericarp (10 × 20), **a** *Oenanthe incrassans* **b** *Oenanthe pimpinelloides*, (e: epidermis, es: endosperm, m: mesocarp, sc: sclerenchyma, t: testa, v: vittae).

Size and shapes of mericarps show morphological differences between the two species. The cross section shape of mericarps is semi-circular in outline and 4–ridged at the dorsal surface in *Oenanthe incrassans*. However, it is triangular in outline and only faintly 4-ridged in *Oenanthe pimpinelloides*. Mesocarp tissue consists of two types of cells; parenchymatic-slightly thickened cells and lignified sclerenchyma cells around vascular bundles. There are 9–10–seriate parenchymatic cells in *Oenanthe incrassans*, but there are 4–5– seriate parenchymatic cells in *Oenanthe pimpinelloides* (Fig. 6a–b).

#### Mericarp micromorphology.

**Figure 7.**
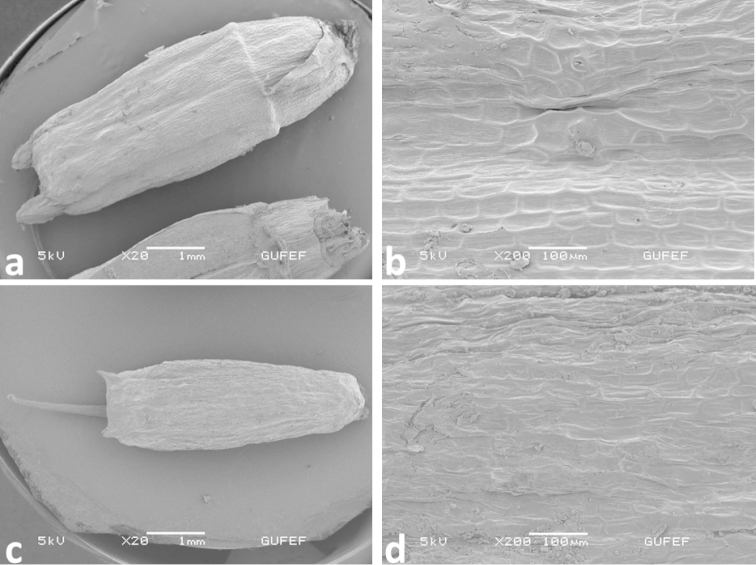
Mericarp microphotography **a–b** *Oenanthe incrassans* (EDG 2098) **c–d** *Oenanthe pimpinelloides* (EDG 2076).

*Oenanthe incrassans* and *Oenanthe pimpinelloides* show fruit characteristics of the genus *Oenanthe*. The fruit micromorphology of *Oenanthe incrassans* differs from *Oenanthe pimpinelloides* by 3.5–4 × 2–2.5 mm sized mericarps (not 2.5–3 × 1–1.5 mm); sepals 0.4–0.9 mm in fruit (not 0.2–0.4 mm); styles shorter than fruit body (not ± equal fruit body); pedicel width ± equal fruit body width (not narrower). While lateral ridges of mericarp are 0.7–0.9 mm width in *Oenanthe incrassans*, it is 0.5–0.6 mm width in *Oenanthe pimpinelloides*. Stylopodium is conical and embedded along calyx line in both species (Fig. 7).

#### Pollen morphology.

**Figure 8.**
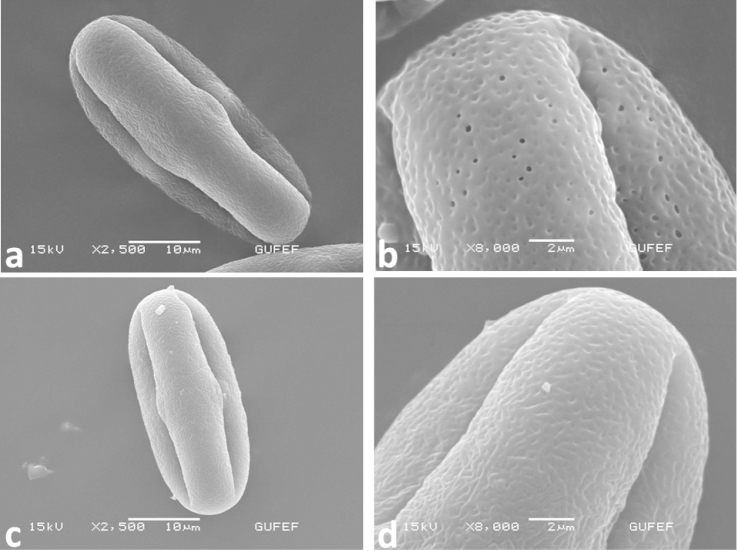
Ornamentation and aperture of pollen by SEM: **a–b** *Oenanthe incrassans* (EDG 2009) **c–d** *Oenanthe pimpinelloides* (EDG 2043).

The pollen grains characters of *Oenanthe incrassans* and *Oenanthe pimpinelloides* are given in the Table 2 for the first time. Umbelliferae is a stenopalynous family (Erdtman 1952). Cerceau- Larrival (1962) divided the pollen of Umbelliferae into 5 types based on P/E ratio: subrhomboidal (type 1, P/E: 1–1.5), subcircular (type 2, P/E: 1–1.5), oval (type 3, P/E: 1.5–2), subrectangular (type 4, P/E: 2), and equatorially constricted (type 5, P/E: over 2). In the present study, pollen of *Oenanthe incrassans* is equatorially constricted (type 5, P/E: over 2) and *Oenanthe pimpinelloides* is oval (type 3, P/E: 1.5–2). According to Erdtman (1943), *Oenanthe incrassans* pollen grains are perprolate (P/E > 2), *Oenanthe pimpinelloides* pollen grains are prolate (P/E: 1.33–2.00). Table 2 shows that pollen size of two species are significantly different. Mature pollen grains of *Oenanthe incrassans* are longer than *Oenanthe pimpinelloides* (Table 1) (Fig. 8).

**Table 2.** Detailed comparison table of pollen features of *Oenanthe incrassans* and *Oenanthe pimpinelloides*.

	*Oenanthe incrassans* (EDG 2009)	*Oenanthe pimpinelloides* (EDG 2028)
Symmetry	Radial, isopolar	Radial, isopolar
Pollen shape (P/E)	Perprolate (P/E = 2.09)	Prolate (P/E = 1.89)
Equatorial outline	Elliptic	Elliptic
Ornamentation	Rugulate (equatorial area), perforate (polar area)	Rugulate(equatorial area), perforate (polar area)
Exine sculpturing	Subtectate	Subtectate
Polar Axis (P) (min-max)	43.2±0.41 mm (37 mm–47 mm)	31.2±0.43 mm (26 mm–36 mm)
E1 = Equatorial Axis (wide side of polen) (min-max)	21.0±0.27 mm (15 mm–22.5 mm)	14.8±0.22 mm (12 mm–17 mm)
E0 = Equatorial Axis (center of pollen) (min-max)	20.6±0.26 mm (15.5 mm–22 mm)	16.5±0.24 mm (13 mm–19 mm)
E2 = Equatorial Axis (narrow side of polen) (min-max)	20.6±0.26 mm (15.5 mm–22 mm)	16.5±0.24 mm (13 mm–19 mm)
Clg = Colpus length (min-max)	33.9±0.44 mm (27 mm–36 mm)	24.3±0.41 mm (20 mm–29 mm)
Clt = Colpus width (min-max)	2.1±0.04 mm (1.5 mm–2.5 mm)	1.9±0.04 mm (1.5 mm–2.5 mm)
Plg = Pore length (min-max)	5.3±0.12 mm (3 mm–6 mm)	4.9±0.10 mm (4 mm–6 mm)
Plt = Pore width (min-max)	5.0±0.07 mm (3 mm–6 mm)	4.9±0.12 mm (4 mm–7 mm)
Int-e = Intine equatorial (min-max)	1.1±0.01mm (1 mm–1.25 mm)	0.9±0.02 mm (0.75 mm–1 mm)
Int-p = Intine polar (min-max)	1.0±0.01 mm (1 mm–1.25 mm)	0.7±0.03 mm (0.50 mm–1 mm)
Ex-e = Exine equatorial (min-max)	1.1±0.02 mm (1 mm–1.25 mm)	0.9±0.02 mm (0.75 mm–1 mm)
Ex-p = Exine polar (min-max)	1.0±0.01 mm (0.75 mm–1.25 mm)	0.7±0.03 mm (0.50 mm–1 mm)

## Discussion

There are many studies about anatomical features of genera of Apiaceae (Liu 2003, Ashena 2014, Akalin Urusak 2013, Yilmaz 2013, Lyskov 2015) because the fruit morphology and anatomy are distinctive characters which frequently clarify the similarities and differences between species. Also a lot of research shows that pollen features of the members of Apiaceae help to distinguish at species (Hebda 1985, Doğan Güner 2011, Yılmaz 2013).

*Oenanthe pimpinelloides* shows wide distribution in the World and also in Turkey. The species has been recorded Aegean, Mediterrenean, Thrace and Black Sea region in Turkey but not the East Anatolian region (Figure 2). Investigation of the collected specimens show that the plant has variable leaf characters. *Oenanthe pimpinelloides* not only prefers wetlands or marshy areas, but is also found on dry slopes or under the shade of trees. It has longer stems and leaves in wetlands than in dry habitats, but its inflorescence and fruit features remain unchanged throughout its range. On the other hand, *Oenanthe incrassans* only occurs in wet areas and it shows similar morphology all localities. Foley and Southam mentioned that morphological characteristics of *Oenanthe incrassans* were retained even in cultivation in England (Foley and Southam 2007). *Oenanthe incrassans* is placed in synonymy of *Oenanthe pimpinelloides* in Hedge and Lammond (1972) because these authors thought some characters such as the thick peduncle and rays of *Oenanthe incrassans* only reflected local variation of *Oenanthe pimpinelloides*. After collecting *Oenanthe incrassans* and a lot of specimens of *Oenanthe pimpinelloides* from different localities, and observing the differences outlined above (Table 1), we agree with treatment of Foley and Southam 2007 and recognize them as distinct species. Therefore, there are nine species *Oenanthe* species in Turkey.

## Supplementary Material

XML Treatment for Oenanthe incrassans

## Appendix

**Selected Specimens Examined.** *Oenanthe pimpinelloides*: **TURKEY. Edirne**: Keşan, Murat Köy Dam Lake, road sides, steppe, 90 m, 19 June 2014, 40°45"5.8'N / 26°45"5.6'E, ED. Güner 2047 & B. Bani (GAZI); **Kırklareli**: Demirköy, clearings forest, 628 m, 18 June 2014, 41°49"27.7'N / 41°46"35.3'E, ED. Güner 2043; ibid 03 August 2014 ED. Güner 2076 & B. Bani (GAZI); **Canakkale**: Biga –Kemer, 90 m, 19 June 2014, 40°24"43.3'N / 27°4"6.9'E, ED. Güner 2049 (GAZI); **Bolu**: Abant-Bolu, Akçaalan village, wet meadows, 918 m, 17 June 2014, 40°39"36.2'N/31°25"01.6'E, ED. Güner 2036 & B. Bani (GAZI); **Ordu**: Fatsa, clearings forest, level, 23 July 2014, ED. Güner 2073 (GAZI); **Rize**: Arşin - Rize, 22 m, 29 June 2014, 40°57"30.5'N / 39°57"11.2'E, ED. Güner 2063 (GAZI); **Muğla**: Çandır, meadows, 5–10 m, 1 June 2014, 36°49"46.0'N / 28°37"54.7'E, ED. Güner 2017 & B. Bani (GAZI).
